# Lymphocyte maintenance during healthy aging requires no substantial alterations in cellular turnover

**DOI:** 10.1111/acel.12311

**Published:** 2015-01-28

**Authors:** Liset Westera, Vera van Hoeven, Julia Drylewicz, Gerrit Spierenburg, Jeroen F van Velzen, Rob J de Boer, Kiki Tesselaar, José A M Borghans

**Affiliations:** 1 Laboratory of Translational Immunology, Department of Immunology, University Medical Center UtrechtLundlaan 6, 3584 EA, Utrecht, The Netherlands; 2 Theoretical Biology and Bioinformatics, Department of Biology, Utrecht UniversityPadualaan 8, 3584 CH, Utrecht, The Netherlands

**Keywords:** healthy aging, homeostasis, lymphocyte turnover, mathematical modeling, stable isotope labeling, thymus involution

## Abstract

In healthy humans, lymphocyte populations are maintained at a relatively constant size throughout life, reflecting a balance between lymphocyte production and loss. Given the profound immunological changes that occur during healthy aging, including a significant decline in T-cell production by the thymus, lymphocyte maintenance in the elderly is generally thought to require homeostatic alterations in lymphocyte dynamics. Surprisingly, using *in vivo* ^2^H_2_O labeling, we find similar dynamics of most lymphocyte subsets between young adult and elderly healthy individuals. As the contribution of thymic output to T-cell production is only minor from young adulthood onward, compensatory increases in peripheral T-cell division rates are not required to maintain the T-cell pool, despite a tenfold decline in thymic output. These fundamental insights will aid the interpretation of further research into aging and clinical conditions related to disturbed lymphocyte dynamics.

## Introduction

Advanced aging is associated with greater susceptibility to infections, reduced vaccine efficacy, and a higher incidence of cancer and autoimmune disease (Goronzy & Weyand, 2013; Montecino-Rodriguez *et al*., 2013). This is believed to be at least partly due to aging of the immune system. Immunological aging is a process characterized by several micro-environmental and cellular changes in the hematopoietic system, which collectively affect both the production and functioning of the peripheral blood lineages. Particularly in the adaptive immune system, profound age-associated changes take place at the cell population level, both in the T-cell and in the B-cell pools.

In the human peripheral T-cell pool, immunological aging is reflected by a numerical decline of naive T cells, loss of T-cell receptor diversity, and changes in T-cell subset distribution (Fagnoni *et al*., 2000; Lazuardi *et al*., 2005; Saule *et al*., 2006; Goronzy *et al*., 2007; Wertheimer *et al*., 2014). The widely held view is that these changes are caused by a combination of lifelong exposure to various pathogens and the gradual involution of the thymus, an irreversible process during which functional thymic tissue becomes progressively replaced by fat (Steinmann *et al*., 1985). Also, the total number of circulating γδ T cells has been reported to decline with age, which is mainly due to a reduction in the most dominant Vδ2 subset (Argentati *et al*., 2002; Michishita *et al*., 2011). The composition of the γδ T-cell population changes with age, with a skewing toward differentiated effector cells in the elderly (Re *et al*., 2005). The observation that absolute γδ T-cell numbers in young adults thymectomized during early childhood were similar to those in healthy controls (Roux *et al*., 2013) suggests that the decline of γδ T cells during healthy aging may be independent of thymic involution. An alternative explanation could, however, be that thymic tissue may have regrown (van Gent *et al*., 2011). Despite large interindividual variation in peripheral B-cell numbers, studies have uniformly reported a numerical decline of total CD19^+^ B cells with age (Ademokun *et al*., 2010; Kogut *et al*., 2012). How different B-cell subsets are affected by aging is less clear, with conflicting literature reporting decreased or unchanged naive B-cell numbers and increased or decreased memory B-cell numbers (Ademokun *et al*., 2010; Kogut *et al*., 2012). There is no unambiguous evidence for declining B-cell production by the bone marrow in humans with age, although an age-related reduction in B-cell progenitors has been reported by a few studies (Ogawa *et al*., 2000; McKenna *et al*., 2001; Kuranda *et al*., 2011) and a pronounced loss of B-cell receptor repertoire diversity was observed in some elderly (Dunn-Walters & Ademokun, 2010), which could reflect decreased bone marrow output.

The occurrence of lymphopenia-induced proliferation in rodents (Miller & Stutman, 1984; Bell *et al*., 1987; Freitas & Rocha, 2000) has suggested that the immune system has an intrinsic capacity to maintain cell numbers at sufficiently high levels by inducing a compensatory homeostatic response when cell numbers are low. Because such responses rely on a general principle of cellular competition for limiting resources [e.g., stimulatory signals from endogenous peptide/MHC complexes and cytokines such as IL-7 (Fry & Mackall, 2001)], it is thought that similar compensatory mechanisms can also be called into action in humans, for example, in response to lymphopenia in HIV infection or following stem cell transplantation (SCT), and in response to thymic involution during aging. Indeed, previous aging studies have reported increased percentages of proliferating Ki-67^+^ naive T cells in the elderly, correlating with reduced naive T-cell pool size, and hence suggestive of a homeostatic increase in cell production triggered by low numbers (Naylor *et al*., 2005; Sauce *et al*., 2012). Aging is, however, a complex, multifactorial process with a highly variable impact on health status, which makes it difficult to determine to what extent chronological age contributes to age-associated changes in the immune system. Here, we selected only individuals with a particularly good health status and used *in vivo* labeling with deuterated water (^2^H_2_O) to quantify the turnover rates of naive, memory, and natural effector B cells, naive and memory CD4^+^ and CD8^+^ T cells, and γδ T cells in young and elderly healthy individuals. In contrast to the analysis of Ki-67 expression, providing a snapshot of the fraction of cells dividing at a single moment, labeling with ^2^H_2_O allowed us to record lymphocyte turnover over a longer period of time, thereby providing a very robust and reliable tool to quantify these dynamics. By combining the parameters obtained by ^2^H_2_O labeling and T-cell receptor excision circle (TREC) analysis in a mathematical model devised previously by den Braber *et al*. (2012), we also quantified to what extent thymic output declines during healthy aging, and whether and how this decline is compensated for by peripheral homeostatic mechanisms. Our data show that the turnover rates of almost all lymphocyte subsets hardly change during healthy aging. Only naive CD8^+^ T cells had a significantly faster turnover in elderly individuals, which was related to a larger fraction of CD95^+^ T cells in older individuals. Despite the observation that CD4^+^ T-cell production by the thymus declines at least tenfold between the third and seventh decade of life, we find no signs of peripheral compensation for this loss of naive T-cell production.

## Results

### Individuals and follow-up

To quantify the dynamics of different leukocyte subsets in healthy aging, five young individuals [plus five from a previous study by Vrisekoop *et al*. (2008)] and ten elderly individuals were enrolled in a heavy water (^2^H_2_O) labeling study (Table1). During a 9-week labeling period, and a subsequent delabeling period of approximately 1 year, we frequently collected blood samples for the measurement of deuterium enrichment in the DNA of granulocytes, B-cell subsets, total γδ T cells, and αβ T-cell subsets (for details on sort gating strategy, see Fig. S1; Supporting information). The average turnover rate (“*p*”), that is, the percentage of a cell population that is replaced by new cells per day, was estimated from the enrichment data using a multi-exponential model, which takes into account that populations can contain cells with different turnover rates (Ganusov *et al*., 2010; Westera *et al*., 2013a). The enrichment curves of all leukocyte subsets were normalized to the estimated maximum level of label incorporation in peripheral blood granulocytes, as this cell population is known to turn over rapidly. The dynamics of granulocytes were similar between young and elderly individuals (Fig. S2; Supporting information).

**Table 1:**
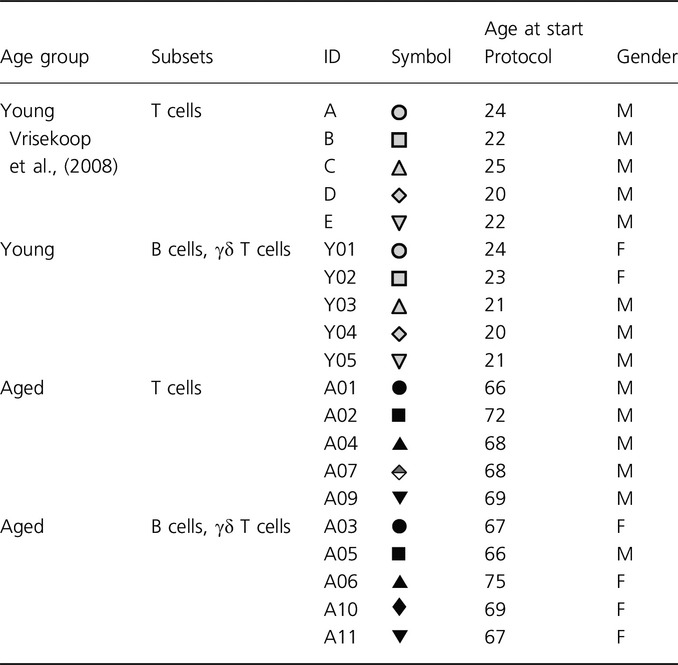
Subject characteristics

### Dynamics of naive, memory, and natural effector B cells

**Figure 1:**
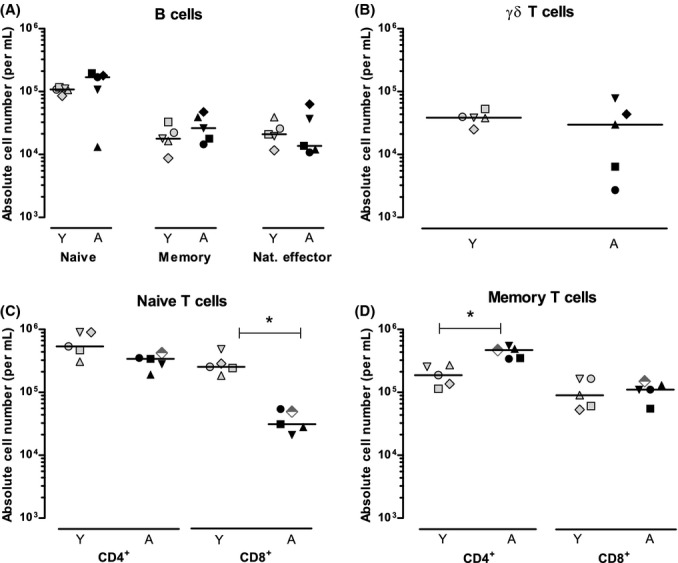
Absolute numbers of B cells and T cells in young and elderly individuals. Absolute numbers (per ml of blood) of (A) naive, memory, and natural effector B cells, (B) γδ T cells, (C) naive CD4^+^ and CD8^+^ T cells, and (D) memory CD4^+^ and CD8^+^ T cells in young (gray symbols) and aged (black symbols) individuals. The elderly male tested seropositive for CMV is depicted by a semi-filled diamond (C+D). Horizontal lines represent median values. Asterisks mark significant differences (*P*-value < 0.05) between young and aged individuals. Different symbols indicate different individuals (see Table1) within panels (A+B) and within panels (C+D); note that different individuals were included for analysis of B-cell subsets and γδ T cells (A+B) and T-cell subsets (C+D).

**Figure 2:**
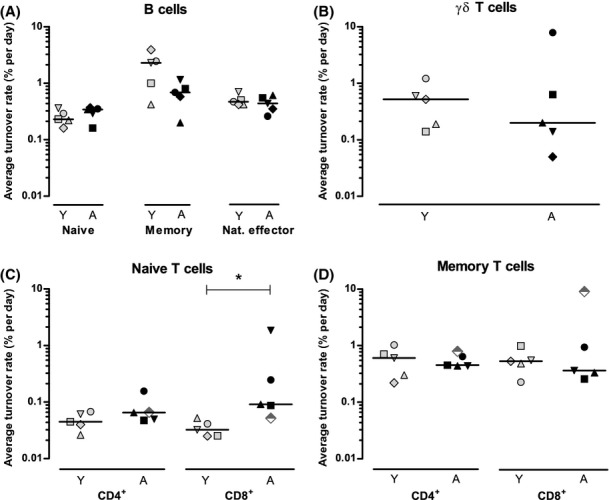
Summary of estimated average turnover rates in young and elderly individuals. Estimates of the average turnover rate of (A) naive, memory, and natural effector B cells, (B) γδ T cells, (C) naive CD4^+^ and CD8^+^ T cells, and (D) memory CD4^+^ and CD8^+^ T cells in young (gray symbols) and aged (black symbols) individuals. The elderly male tested seropositive for CMV is depicted by a semi-filled diamond (C+D). All estimates were obtained by fitting the multi-exponential model to the individual data sets (see Supporting Information). Horizontal lines represent median values. The asterisk marks a significant difference (*P*-value < 0.05) between young and aged individuals. Individual fits are shown in Figs S3–S5. Different symbols indicate different individuals within panels (A+B) and panels (C+D).

To investigate whether aging is associated with alterations in peripheral B-cell dynamics, we first determined the absolute number and distribution of the three main B-cell subsets present in the circulation, that is, naive (IgM^+^CD27^−^), memory (IgM^−^CD27^+^), and natural effector (IgM^+^CD27^+^) B cells. For none of the subsets we found significant age-related differences in absolute B-cell counts, although the interindividual variation in naive B-cell numbers in the aged was relatively large (Fig.1A, Table2). As there are indications that B-cell production by the bone marrow declines with age (Ogawa *et al*., 2000; McKenna *et al*., 2001; Kuranda *et al*., 2011), B-cell numbers may stay constant because of compensatory changes in peripheral B-cell dynamics. Fitting the multi-exponential model to the enrichment data of each individual (Figs S3 and S4; Supporting information) revealed that the average turnover rates of all B-cell subsets in elderly individuals were not significantly different from those in young individuals (Fig.2A, Table2). The interindividual variation in memory B-cell turnover rates between young subjects was rather large, which was not related to the relative abundance of the different B-cell subsets in these individuals. In conclusion, variation in B-cell dynamics between individuals seemed to be related to factors other than aging.

**Table 2:** Median (range) of average turnover rates, cell numbers, and total daily production of the different lymphocytes

	Average turnover rate (% per day) median (range)		Cell number (per μl blood) median (range)		Total production (×10^6^ cells per day) median (range)	
	Young	Aged	Young	Aged	Young	Aged
Naive B cells	0.23 (0.16–0.36)	0.34 (0.16–0.37)	108 (85–117)	170 (13–196)	67 (33–101)	78 (11–167)
Mem B cells	2.29 (0.42–3.89)	0.69 (0.20–1.15)	18 (9–33)	26 (14–47)	85 (17–136)	36 (19–75)
Nat Eff B cells	0.47 (0.42–0.70)	0.44 (0.26–0.61)	21 (12–39)	14 (11–63)	31 (12–41)	19 (7–54)
γδ T cells	0.52 (0.14–1.21)	0.20 (0.05–7.86)	38 (25–53)	30 (3–78)	33 (18–120)	15 (5–53)
Naive CD4^+^ T cells	0.04 (0.03–0.07)	0.07 (0.05–0.16)	534 (307–898)	337 (192–417)	89 (20–137)	40 (31–138)
Naive CD8^+^ T cells	0.03 (0.03–0.05)	0.09 (0.05–1.86)	254 (185–484)	31 (21–54)	24 (15–39)	7 (6–98)
Mem CD4^+^ T cells	0.60 (0.22–1.02)	0.45 (0.44–0.80)	403 (221–448)	470 (339–546)	391 (221–911)	542 (393–936)
Mem CD8^+^ T cells	0.53 (0.23–0.98)	0.36 (0.26–9.12)	90 (53–165)	113 (55–153)	109 (70–227)	107 (35–3497)

### Dynamics of γδ T cells and naive and memory CD4^+^ and CD8^+^ T cells

We compared absolute numbers of naive (CD27^+^CD45RO^−^) and memory (CD45RO^+^) CD4^+^ and CD8^+^ αβ T cells and γδ T cells in young and elderly individuals (Fig.1B,C,D, Table2). Naive T-cell numbers tended to be lower in elderly subjects; this difference was significant for CD8^+^ (*P*-value = 0.008; Fig.1C) but not for CD4^+^ (*P*-value = 0.06) naive T cells. The number of memory CD4^+^ T cells was significantly higher in elderly subjects (*P*-value = 0.008), whereas the number of memory CD8^+^ T cells was not different in young and aged individuals (Fig.1D). We observed considerable interindividual variation in the number of γδ T cells, but did not find a significant difference between the age groups (Fig.1B). Within the γδ T-cell pool, the fraction of Vδ2^+^ cells was not different in young and elderly subjects (median values were 40% for young and 57% for elderly individuals).

As changes in the size of the T-cell subsets might be related to changes in their dynamics, we quantified their turnover rates. For γδ T cells, fitting the multi-exponential model to the deuterium-enrichment data (Figs S3 and S4) yielded no different average turnover rates between young and aged individuals (Fig.2B, Table2). For memory T cells, the average turnover rates estimated from the enrichment data [(Westera *et al*., 2013a) and Fig. S5; Supporting information] were also not different between young and elderly subjects (Fig.2D, Table2). Hence, the age-related increase in memory CD4^+^ T-cell numbers was not concomitant with altered turnover rates.

**Figure 3:**
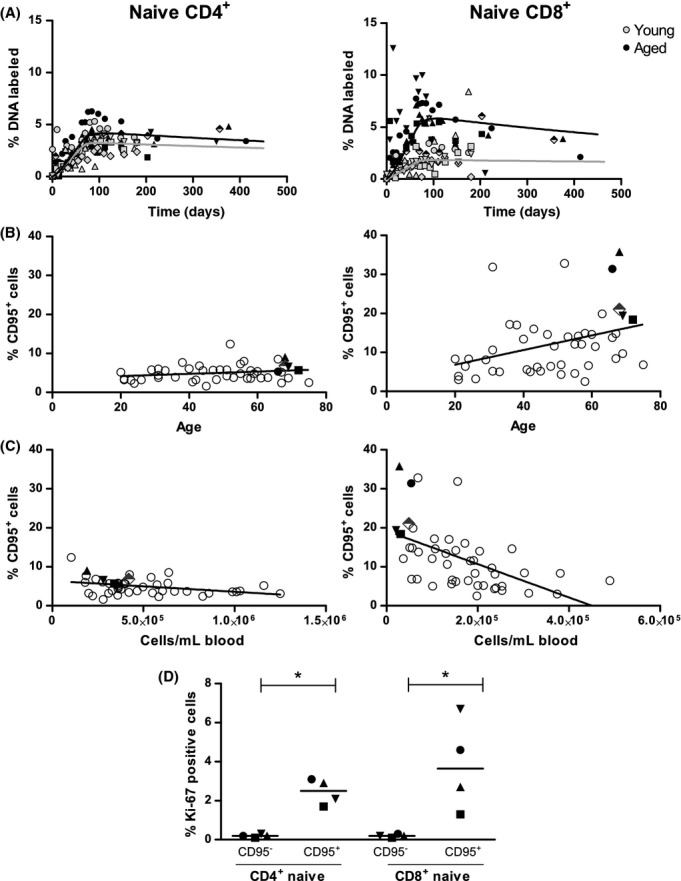
Analysis of ^2^H enrichment and CD95 expression within the naive T-cell pools. (A) Best fits of the mixed effect multi-exponential model (see Data S1; Supporting information) to ^2^H enrichment in the DNA of naive CD4^+^ and CD8^+^ T cells from young [(Vrisekoop *et al*., 2008), gray symbols and curve] and elderly individuals (black symbols and curve). Label enrichment was scaled between 0 and 100% by normalizing for the maximum enrichment in granulocytes. (B, C) The expression of CD95 on naive CD4^+^ (left) and CD8^+^ (right) T cells was determined in elderly males (black symbols) of the ^2^H_2_O labeling study and in other healthy donors of varying ages (open symbols, n = 41). The percentage of CD95^+^ naive T cells was plotted against age (B) and against the number of naive CD4^+^ or CD8^+^ T cells per ml blood (C). The elderly male who tested seropositive for CMV is depicted by a semi-filled diamond (B+C). The lines in panels (C) and (D) represent linear regression analyses. (D) Ki-67 expression was measured within the CD95^−^ and CD95^+^ fractions of naive CD4^+^ and CD8^+^ T cells in elderly males of our labeling study (n = 4). The median is represented by a horizontal line. Different symbols indicate different individuals. Asterisks mark significant differences (*P*-value < 0.05).

Deuterium enrichment in naive T cells tended to be higher in elderly than in young individuals (Fig.3A), especially in the case of naive CD8^+^ T cells, suggesting a faster turnover for this subset in elderly individuals. Fitting the multi-exponential model to the enrichment data of each individual (Fig. S5) revealed that the average turnover rate of naive CD4^+^ T cells was not significantly different between the age groups (median *p*_*young*_ = 0.04% and *p*_*aged*_ = 0.07% per day, *P*-value = 0.2), whereas the average turnover rate of naive CD8^+^ T cells was significantly higher in elderly subjects (median *p*_*young*_ = 0.03% and *p*_*aged*_ = 0.09% per day, *P*-value = 0.02; Fig.2C, Table2). Even though this increase should be interpreted with caution in light of the small group sizes and the large interindividual variation in the elderly group, the data show that the turnover rate of naive CD8^+^ T cells in four of the five elderly individuals was higher than in the young (Fig.2C).

### Turnover-associated changes in the naive CD8^+^ T-cell pool in elderly subjects

To study whether the increased turnover of naive CD8^+^ T cells concurred with other alterations within this subset, we analyzed the naive T-cell pools of the elderly subjects in more detail. Because samples of the young subjects who received ^2^H_2_O were no longer available, we also analyzed the composition of the naive CD8^+^ T-cell pool in 41 additional healthy controls of different ages. In both young and elderly individuals, naive CD4^+^ and CD8^+^ T cells had a high expression of CCR7 and CD28 (median values for naive CD4^+^: 98% CCR7^+^ and 98% CD28^+^; for naive CD8^+^: 95% CCR7^+^ and 96% CD28^+^), confirming their naive phenotype. However, we found substantial age-related differences in the percentage of naive CD8^+^ T cells expressing CD95 (Fig.3B). Whereas naive CD4^+^ T cells had a low expression of CD95 at any age (generally around 5% CD95^+^), the percentage of naive CD8^+^ T cells expressing CD95 increased over age, comprising between 18% and 36% in the elderly subjects (Fig.3B), and appeared to be inversely correlated with the number of naive T cells (for CD4^+^: *r* = −0.37, *P*-value = 0.01; for CD8^+^: *r* = −0.54, *P*-value < 0.0001; Fig.3C). To investigate whether the relatively large CD95^+^ fraction of naive CD8^+^ T cells in elderly subjects could have contributed to the faster turnover rate of the aged naive CD8^+^ T-cell pool, we measured the expression of the cell-cycle marker Ki-67 in the CD95^−^ and CD95^+^ fractions of the naive CD4^+^ and CD8^+^ T-cell pools. Indeed, the percentage of Ki-67-expressing cells was significantly higher among CD95^+^ compared to CD95^−^ cells (*P*-value = 0.03 for both CD4^+^ and CD8^+^; Fig.3D). Thus, the larger fraction of rapidly proliferating CD95^+^ cells could explain the observed increase in turnover of the ‘naive’ CD8^+^ T-cell pools of the elderly individuals.

### No peripheral homeostatic compensation in the naive CD4^+^ T-cell pool, despite decreasing thymic output

**Figure 4:**
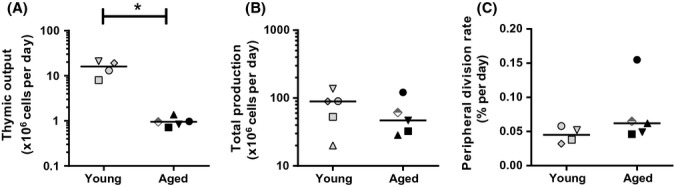
Contributions of daily thymic output and peripheral T-cell division to total naive CD4^+^ T-cell production. (A) Estimated daily thymic output (cells per day) in young and aged individuals, calculated by multiplying the total daily naive CD4^+^ T-cell production by the normalized naive CD4^+^ T-cell TREC content (normalized using TREC contents of single positive CD4^+^ thymocytes), as described previously (den Braber *et al*., 2012). The asterisk marks a significant difference (*P*-value < 0.05) in daily thymic output between young and aged individuals. (B) Total production of naive CD4^+^ T cells per day in young and aged individuals, calculated as (the average turnover rate *p*) × (the absolute number of naive CD4^+^ T cells per liter blood) × (5 L blood) × 50, assuming that 2% of lymphocytes reside in the blood (Westermann & Pabst, 1990). (C) Estimated daily peripheral division rate per cell, calculated by dividing the estimated total peripheral T-cell division (in Fig. S7B) by the total number of naive CD4^+^ T cells present in the periphery. The normalized TREC content of naive CD4^+^ T cells in young individuals was previously reported in den Braber *et al*. (2012); as no TREC measurements were obtained for individual C, this individual was not included in the analysis. TREC contents are shown in Fig. S7A. Different symbols indicate different individuals. The elderly male who tested seropositive for CMV is depicted by a semi-filled diamond.

Because the thymus involutes with age (Steinmann *et al*., 1985), and deuterium is incorporated by new naive T cells that are produced in both the thymus and the periphery, the similar turnover rates of naive CD4^+^ T cells in our young and elderly individuals could be an indication for a compensatory increase in peripheral T-cell division in the elderly. Therefore, we quantified the contribution of thymic T-cell production and peripheral T-cell division to the daily turnover of naive CD4^+^ T cells in young and elderly subjects. We previously demonstrated that daily thymic output can be deduced from the average turnover rate, the absolute cell number, and the TREC content of naive T cells (den Braber *et al*., 2012). Using this approach (see Supporting Information), we estimated that thymic output declined significantly from 16 million cells per day in young individuals to < 1 million cells per day in elderly individuals (*P*-value = 0.02; Fig.4A), a change that is well in line with the previously estimated tenfold decrease in thymic output during adulthood (Steinmann *et al*., 1985). By subtracting the estimated daily thymic output from the total daily production of naive CD4^+^ T cells (Fig.4B), we deduced the average naive CD4^+^ peripheral T-cell division rate in young and elderly subjects (see Supporting Information). Remarkably, despite the tenfold decrease in thymic output, peripheral naive CD4^+^ T-cell division rates were not significantly higher in the elderly (Fig.4C), suggesting that peripheral homeostatic compensation for loss of thymic output either did not occur or was negligible.

## Discussion

Using long-term *in vivo* ^2^H_2_O labeling in healthy young and elderly individuals, we observed neither age-related differences in population dynamics nor signs of compensatory mechanisms for the population maintenance of different B-cell and T-cell subsets. Our data convincingly point out that maintenance of lymphocyte populations during healthy aging does not require substantial alterations in lymphocyte turnover. These results are consistent with a previous deuterated glucose labeling study, which reported no significant age-related difference in the turnover of total B cells (Macallan *et al*., 2005), and we extend these insights by showing that also within the different B-cell subsets as well as for γδ T cells, turnover rates do not change during healthy aging.

Although lymphopenia-induced T-cell proliferation is clearly triggered in rodents with low T-cell numbers (Miller & Stutman, 1984; Bell *et al*., 1987; Freitas & Rocha, 2000), there is no unambiguous evidence for the occurrence of homeostatic T-cell proliferation in primates and humans. Increased percentages of Ki-67-expressing T cells have been observed in different clinical conditions of lymphopenia, including in HIV infection, after SCT, and post-thymectomy (Hazenberg *et al*., 2000b, 2002; Borghans *et al*., 2006; van Gent *et al*., 2011), and also under more physiological circumstances in aging rhesus macaques and humans (Naylor *et al*., 2005; Cicin-Sain *et al*., 2007; Sauce *et al*., 2012). In the rhesus macaque model of immune senescence, fractions of Ki-67^+^ naive T cells were found to correlate positively with age, and negatively with the percentage of naive cells in the CD4^+^ and CD8^+^ T-cell pools and with TCR diversity (Cicin-Sain *et al*., 2007). Naive T-cell turnover rates increased exponentially when the percentage of naive T cells in the CD8^+^ T-cell pool dropped below 4% (Cicin-Sain *et al*., 2007), supporting the idea that a certain pool size threshold may exist below which compensatory mechanisms get activated. In humans, Naylor *et al*. (2005) reported an increase in CD4^+^ T-cell division rates after the age of 70, and Sauce *et al*. (2012) observed a direct association between decreased naive T-cell numbers and increased frequencies of Ki-67^+^ naive T cells in healthy elderly individuals aged 76 and older. Although such correlations may be suggestive for the occurrence of homeostatic proliferation, it is in fact not clear whether increased cell division rates are induced by low cell numbers. What may be interpreted as a favorable homeostatic response to low cell numbers may alternatively reflect a different, perhaps even maleficent proliferative process. In fact, a third factor (related to aging) may induce both cell loss and increased lymphocyte turnover, or increased lymphocyte proliferation could even be *driving* cell loss. Increased levels of proliferation observed in HIV and SCT patients, for example, turned out to be related to immune activation or clinical events, rather than to reflect a homeostatic response to low cell numbers (Hazenberg *et al*., 2000b, 2002). Likewise, a chronic inflammatory state associated with aging (Macaulay *et al*., 2013) may drive increased lymphocyte proliferation and lymphocyte loss.

We found a reduced pool size and an increased turnover rate of naive CD8^+^ T cells in the aged, which was accompanied by the relative abundance of cycling CD95^+^ T cells. As expression of CD95 has been shown to be upregulated in response to IL-7 *in vitro* (Cimbro *et al*., 2012), and IL-7 is known to play a key role in regulating proliferative responses *in vivo* (Takada & Jameson, 2009), these CD95^+^ cells could in theory reflect homeostatically dividing naive CD8^+^ T cells. However, this idea is not supported by the observation that almost all CD95^+^ cells expressed the IL-7 receptor (> 90% CD127^+^), which is typically downregulated upon IL-7 binding. Phenotype analyses indicated that the CD95^+^ (CD27^+^CD45RO^−^) CD8^+^ T-cell population contained both memory stem cells (Gattinoni *et al*., 2011), expressing CCR7 and CD28, and effector-like (CCR7^−^CD28^+/−^) cells (for representative density dot plots, see Fig. S6; Supporting information). These results stress that the age-related increase in naive CD8^+^ T-cell turnover should not be interpreted as evidence for homeostatically increased naive CD8^+^ T-cell division at old age.

Deuterium labeling data of the naive CD4^+^ T-cell pool gave more straightforward insights into the possible role of homeostatic compensation during healthy aging, as this population did not contain high levels of CD95^+^ T cells at any age. We found that the average turnover rate of CD45RA^+^CD27^+^ naive CD4^+^ T cells did not change during healthy aging, which is in line with a previous deuterated glucose labeling study (Wallace *et al*., 2004) which reported similar dynamics of CD45RA^+^ CD4^+^ T cells in healthy young and aged individuals. Remarkably, despite the significant loss of thymic output that we estimated between the 3rd and the 7th decade of life, peripheral naive CD4^+^ T-cell division rates were not increased. Naive CD4^+^ T-cell numbers tended to be reduced in the elderly, but this was not significant (Fig.1C). The significant drop in naive CD8^+^ T-cell numbers and the nonsignificant change in naive CD4^+^ T-cell numbers over age are perfectly in line with observations from a recent large cross-sectional study, in which aging correlated with a decline in the naive CD4^+^ count in CMV-positive individuals, but not in CMV-negative individuals (Wertheimer *et al*., 2014). In this respect, it is important to note that nine of the ten healthy elderly individuals in our study were CMV-negative (the only CMV-positive elderly individual is marked in all figures by a semi-filled diamond). Loss of thymic output was also not compensated for by increased cell survival, as this should have been reflected by reduced naive T-cell turnover rates. Hence, our data show no signs of homeostatic compensation for reduced thymic output in the naive CD4^+^ T-cell pool during healthy aging.

As deuterium labeling studies are always limited to relatively small sample sizes, one may wonder whether our study had the power to detect compensatory responses in lymphocyte turnover during healthy aging. Indeed, statements of statistical nonsignificance should be interpreted with caution for such small sample sizes. Our study was, however, able to detect twofold changes in lymphocyte turnover with a power larger than 80% and 1.5-fold changes with a power larger than 70%. We show that the most likely explanation for our findings is that thymic output contributes so little to the total production of naive T cells even in young adults (Fig. S7C; Supporting information) and that a compensatory response for the further decline in thymic output with age is simply not required or too small to be measured. Importantly, the disadvantage of small numbers of individuals in deuterium labeling studies is counterbalanced by the advantage that they provide very reliable quantitative estimates. This is firstly owing to the frequent sampling per individual during labeling and delabeling phases, and secondly because in long-term labeling studies the information on cell turnover is recorded over a period of several weeks, thereby providing a turnover estimate that is considerably less sensitive to fluctuations in cell turnover than, for example, a ‘snapshot’ measurement of Ki-67 expression.

The lack of correlation between naive CD4^+^ T-cell numbers and turnover rates that we found (Fig. S8; Supporting information) contrasts the previously observed correlations between the fraction of Ki-67^+^ naive T cells and the naive T-cell pool size in elderly rhesus macaques and humans (Naylor *et al*., 2005; Cicin-Sain *et al*., 2007; Sauce *et al*., 2012). Although we cannot formally exclude the possibility that such a correlation may have gone unnoticed because of our relatively small group sizes, we think that other reasons underlie this difference. One option is that age differences explain the contrast between the studies, as our elderly individuals were slightly younger than the subjects in the previous human aging studies (Naylor *et al*., 2005; Sauce *et al*., 2012). However, we think it is more likely that other factors related to immune status underlie the previously observed correlation between T-cell counts and proliferation rates at high age. Because Ki-67 can be expressed by homeostatically dividing cells but also by naive T cells that proliferate to become memory cells, the increased Ki-67 levels observed in some elderly might reflect increased immune activation, for example, due to persistent infection with CMV, which has a high prevalence in the elderly, or to other factors that increase inflammation with age (Macaulay *et al*., 2013). The increased naive T-cell turnover rates observed in aging rhesus macaques (Cicin-Sain *et al*., 2007) are compatible with this scenario, as all monkeys turned out to be CMV-positive (Dr. J. Nikolich-Zugich, personal communication). Although the number of CMV-positive elderly individuals was too low to investigate the effect of CMV in our elderly cohort, the low frequency of CMV-positive individuals among our elderly subjects is at least suggestive that CMV may play a role. Future studies among CMV-positive and CMV-negative individuals are needed to address the role of CMV in truly healthy aging. Finally, although the largely CMV-negative group of elderly individuals included in this study may not be representative for the elderly population, it provided us with the unique opportunity to study whether homeostatic mechanisms are evident in truly healthy aging in the absence of CMV as a possible confounder.

Thanks to the combination of deuterium labeling data and TREC analyses, we were also able to calculate how the *absolute* number of cells produced by the thymus per day changed during healthy aging. We found that thymic output declined from 16 million cells per day in young adults to < 1 million cells per day in elderly individuals, in line with the previously estimated tenfold decrease in thymic output based on histological studies (Steinmann *et al*., 1985). Previously, Bains *et al*. (2009) also combined different techniques to estimate daily thymic output in young adults, by analyzing TREC contents and Ki-67 expression data. Remarkably, with roughly 350 million newly produced naive CD4^+^ T cells per day, their estimate of thymic output was an order of magnitude higher than our estimated 16 million cells per day. Recent work suggests that Bains *et al*. may have overestimated the fraction of dividing cells by the measurement of Ki-67 expression, which appears to remain elevated for days after completion of cell division (de Boer & Perelson, 2013; Hogan *et al*., 2013) and may thereby have indirectly overestimated daily thymic output.

In summary, we have provided reliable estimates of the average turnover rates of various B-cell and T-cell subsets in healthy young and elderly individuals and found no signs of homeostatic compensation during *truly* healthy aging. Our insights will aid the interpretation of past, current, and future investigations in a variety of interventions and diseases, which may reveal, for example, whether increased cell division rates in certain lymphopenic conditions reflect a favorable compensatory mechanism or rather the detrimental effect of inflammation.

## Experimental procedures

### Subjects and *in vivo* ^2^H_2_O labeling

Five young and ten elderly healthy volunteers (Table1) were enrolled in the study after having provided written informed consent. On day 1, volunteers received an oral ramp-up dose of 7.5 ml of ^2^H_2_O (99.8% enriched, Cambridge Isotope Laboratories, Tewksbury, MA, USA) per kg body water, in small portions throughout the day. Body water was assumed to be 60% (males) and 50% (females) of body weight (Watson *et al*., 1980). Blood was drawn before the first portion, and urine was collected after the last portion. As maintenance dose, volunteers drank 1.25 ml kg^−1^ body water at home daily for the duration of the labeling period (9 weeks; for logistic reasons the labeling period was ∽7.5 weeks and ∽10 weeks for two subjects). Urine was collected an additional 15 times during the first ∽100 days of the study. Blood was drawn six more times during labeling and eight times during delabeling, with the last withdrawal ∽1 year after stop of ^2^H_2_O administration. All volunteers were healthy and did not take drugs (a questionnaire was taken to confirm that subjects were healthy and did not have serious illnesses (e.g., malaria; cancer) in the past; serological testing was performed to exclude infection with HIV, HBV, and HCV). To determine CMV serostatus, CMV-specific IgG antibodies were determined in plasma by ELISA according the to manufacturer's instructions (IBL International GmbH). For the purpose of analyzing the T-cell compartment, in particular CD95 expression on naive T cells, additional blood samples were specifically collected from healthy volunteers not following the labeling protocol after having provided written informed consent. This study was approved by the medical ethical committee of the University Medical Center Utrecht and conducted in accordance with the Helsinki Declaration of 1975, revised in 2008.

### Cell isolation, flow cytometry, and sorting

Peripheral blood mononuclear cells were obtained by Ficoll-Paque (GE Healthcare, Little Chalfont, UK) density gradient centrifugation from heparinized blood. Granulocytes were obtained by erythrocyte lysis of the granulocyte/erythrocyte layer. Total peripheral blood mononuclear cells were frozen as a sample with baseline enrichment on the first study day (*t* = 0).

Absolute cell numbers were determined using TruCOUNT tubes (BD Biosciences, San Jose, CA, USA), in which whole blood was stained using CD45-PerCP, CD3-FITC (BioLegend, San Diego, CA, USA), CD8-V500 (BD Biosciences), CD4-APC-eF780, and CD19-eFluor450 (eBioscience, San Diego, CA, USA). After erythrocyte lysis with FACS Lysing Solution (BD Biosciences), tubes were instantly analyzed.

CD95 expression on CD27^+^CD45RO^−^ naive T cells was measured using CD3-eFluor450, CD27-APCeFluor780 (eBioscience), CD8-PerCP (BioLegend), CCR7-APC (R&D systems, Minneapolis, MN, USA), CD45RO-PE-Cy7, CD95-APC, and CD28-FITC (BD Biosciences). To analyze the expression of cell-cycle marker Ki-67, cells were stained with extracellular markers [CD3-eFluor450, CD4-APCeFluor780 (eBioscience), CD8-PerCP, CD27-PE (BioLegend), CD45RO-PE-Cy7, and CD95-APC (BD Biosciences)], fixed and permeabilized (Cytofix/Cytoperm; BD Biosciences), and stained intracellularly with Ki-67-FITC (DAKO, Glostrup, Denmark). Washing steps were performed using Perm/Wash buffer (BD Biosciences). Absolute numbers of cell subsets (e.g., CD95^+^ naive) were calculated using the absolute number of CD4^+^, CD8^+^ T cells, or CD19^+^ B cells from TruCount analysis. All cells were analyzed on an LSR-II flow cytometer using FACSdiva software (BD Biosciences).

For sorting of T-cell subsets, cells were incubated with CD3-FITC, CD4-Pacific Blue, CD8-PerCP-Cy5.5, CD45RO-PE (BioLegend), and CD27-APC (eBioscience). For sorting of B-cell subsets and γδ T cells, cells were incubated with CD3-eFluor450, CD27-APC (eBioscience), CD19-PerCP (BioLegend), F(ab′)2 IgM-FITC (Southern Biotech, Birmingham, AL, USA), and TCR-pan-γδ-PE (Beckman Coulter, Brea, CA, USA). Naive (CD27^+^CD45RO^−^) and memory (CD45RO^+^) CD4^+^ and CD8^+^ T cells, or naive (IgM^+^CD27^−^), memory (IgM^−^CD27^+^), and natural effector (IgM^+^CD27^+^) CD19^+^ B cells and panγδ^+^ T cells were sorted on a FACSAria II cell sorter (BD Biosciences). Flow cytometric analysis and sorting were always performed on freshly isolated cells. Representative density dot plots and the gating strategy for all sorted subsets are shown in Fig. S1.

### DNA isolation

Genomic DNA was isolated from granulocytes, total peripheral blood mononuclear cells (*t* = 0), and sorted cells using the Blood QuickPure kit (Macherey-Nagel, Dueren, Germany) or the Reliaprep Blood gDNA Miniprep System (Promega, Madison, WI, USA) and stored at −20 °C before processing for gas chromatography/mass spectrometry (GC/MS).

### TREC analysis

In sorted naive CD4^+^ T-cell samples of elderly individuals, signal joint TREC numbers and DNA input were quantified with a ViiA™ 7 Real-Time PCR System (Applied Biosystems, Foster City, CA, USA) and calculated as described previously (Hazenberg *et al*., 2000a).

### Measurement of deuterium enrichment in body water and DNA

Deuterium enrichment in DNA from granulocytes and sorted T-cell fractions was measured according to the method described by Busch *et al*. (2007) with minor modifications. Briefly, DNA was enzymatically hydrolyzed into deoxyribonucleotides and derivatized to penta-fluoro-triacetate before injection (DB-17MS column; Agilent Technologies, Santa Clara, CA, USA) into the gas chromatograph (7890A GC System; Agilent Technologies). Penta-fluoro-triacetate was analyzed by negative chemical ionization mass spectrometry (5975C inert XL EI/CI MSD with Triple-Axis Detector; Agilent Technologies) measuring ions m/z 435 and m/z 436. For quantification of ^2^H enrichment, standard solutions with known enrichment (Tracer-to-Tracee ratios ([M + 1]/[M + 0]) 0, 0.0016, 0.0032, 0.0065, 0.0131, 0.0265, 0.0543, and 0.1140) were made by mixing 1-^13^C-deoxyadenosine (Cambridge Isotopes Inc.; generates an ‘M + 1′ ion) with unlabeled deoxyadenosine (Sigma, St. Louis, MO, USA). To correct for abundance sensitivity of isotope ratios, we followed the approach proposed by (Patterson *et al*., 1998) on log 10-transformed enrichment data. Deuterium enrichment in urine was analyzed on the same GC/MS system (using a PoraPLOT Q 25 9 0.32 column; Varian Medical Systems, Palo Alto, CA, USA) by electron impact ionization as previously described (Westera et al., 2013bb).

### Statistical analyses

Our study was designed to detect a twofold or greater difference between groups with a power of 80%, based on a two-sided test with an error α of 5%. The power calculation was based on our previous deuterium labeling studies among healthy young individuals (Vrisekoop *et al*., 2008), which revealed a mean turnover of naive T cells of 0.00042 per day with a SD of 0.00015 per day and a mean turnover of memory T cells of 0.0056 per day with a standard deviation of 0.0028 per day, assuming similar standard deviations at old age. Of note, with the group sizes that were used, even 50% differences should be detected with a power larger than 70%.

Medians were compared between age groups using Mann–Whitney tests (graphpad Software, Inc, La Jolla, CA, USA). Differences with a *P*-value < 0.05 were considered significant. Correlations were analyzed using Pearson's correlation coefficient. Deuterium-enrichment data were fitted with the functions nlme and nlm in r. The 95% confidence intervals were determined using a bootstrap method where the residuals to the optimal fit were resampled 500 times.

## Supporting Information

**Fig. S1** Sort gating strategy. **Fig. S2** Estimated average turnover rates of granulocytes in young and elderly individuals. **Fig. S3** Best fits of ^2^H enrichment in B-cell subsets and in γδ T cells in young individuals. **Fig. S4** Best fits of ^2^H enrichment in B-cell subsets and in γδ T cells in elderly individuals. **Fig. S5** Best fits of ^2^H enrichment in T-cell subsets in elderly individuals. **Fig. S6** Composition of the CD95^+^ fraction of the naive CD8^+^ T-cell pool. **Fig. S7** TREC content and total peripheral division of naive CD4^+^ T cells in young and elderly individuals. **Fig. S8** No correlation between the absolute number and the turnover rate of CD4^+^ naive T cells. **Data S1** Supporting methods.

## References

[b1] Ademokun A, Wu YC, Dunn-Walters D (2010). The ageing B cell population: composition and function. Biogerontology.

[b2] Argentati K, Re F, Donnini A, Tucci MG, Franceschi C, Bartozzi B, Bernardini G, Provinciali M (2002). Numerical and functional alterations of circulating gammadelta T lymphocytes in aged people and centenarians. J. Leukoc. Biol.

[b3] Bains I, Thiebaut R, Yates AJ, Callard R (2009). Quantifying thymic export: combining models of naive T cell proliferation and TCR excision circle dynamics gives an explicit measure of thymic output. J. Immunol.

[b4] Bell EB, Sparshott SM, Drayson MT, Ford WL (1987). The stable and permanent expansion of functional T lymphocytes in athymic nude rats after a single injection of mature T cells. J. Immunol.

[b5] de Boer RJ, Perelson AS (2013). Quantifying T lymphocyte turnover. J. Theor. Biol.

[b6] Borghans JA, Bredius RG, Hazenberg MD, Roelofs H, Jol-van der Zijde EC, Heidt J, Otto SA, Kuijpers TW, Fibbe WE, Vossen JM, Miedema F, van Tol MJ (2006). Early determinants of long-term T-cell reconstitution after hematopoietic stem cell transplantation for severe combined immunodeficiency. Blood.

[b7] den Braber I, Mugwagwa T, Vrisekoop N, Westera L, Mögling R, de Boer AB, Willems N, Schrijver EH, Spierenburg G, Gaiser K, Mul E, Otto SA, Ruiter AF, Ackermans MT, Miedema F, Borghans JA, de Boer RJ, Tesselaar K (2012). Maintenance of peripheral naive T cells is sustained by thymus output in mice but not humans. Immunity.

[b8] Busch R, Neese RA, Awada M, Hayes GM, Hellerstein MK (2007). Measurement of cell proliferation by heavy water labeling. Nat. Protoc.

[b9] Cicin-Sain L, Messaoudi I, Park B, Currier N, Planer S, Fischer M, Tackitt S, Nikolich-Zugich D, Legasse A, Axthelm MK, Picker LJ, Mori M, Nikolich-Zugich J (2007). Dramatic increase in naive T cell turnover is linked to loss of naive T cells from old primates. Proc. Natl Acad. Sci. USA.

[b10] Cimbro R, Vassena L, Arthos J, Cicala C, Kehrl JH, Park C, Sereti I, Lederman MM, Fauci AS, Lusso P (2012). IL-7 induces expression and activation of integrin α4β7 promoting naive T-cell homing to the intestinal mucosa. Blood.

[b11] Dunn-Walters DK, Ademokun AA (2010). B cell repertoire and ageing. Curr. Opin. Immunol.

[b12] Fagnoni FF, Vescovini R, Passeri G, Bologna G, Pedrazzoni M, Lavagetto G, Casti A, Franceschi C, Passeri M, Sansoni P (2000). Shortage of circulating naive CD8(+) T cells provides new insights on immunodeficiency in aging. Blood.

[b13] Freitas AA, Rocha B (2000). Population biology of lymphocytes: the flight for survival. Annu. Rev. Immunol.

[b14] Fry TJ, Mackall CL (2001). Interleukin-7: master regulator of peripheral T-cell homeostasis?. Trends Immunol.

[b15] Ganusov VV, Borghans JA, de Boer RJ (2010). Explicit kinetic heterogeneity: mathematical models for interpretation of deuterium labeling of heterogeneous cell populations. PLoS Comput. Biol.

[b16] Gattinoni L, Lugli E, Ji Y, Pos Z, Paulos CM, Quigley MF, Almeida JR, Gostick E, Yu Z, Carpenito C, Wang E, Douek DC, Price DA, June CH, Marincola FM, Roederer M, Restifo NP (2011). A human memory T cell subset with stem cell-like properties. Nat. Med.

[b17] van Gent R, Schadenberg AW, Otto SA, Nievelstein RA, Sieswerda GT, Haas F, Miedema F, Tesselaar K, Jansen NJ, Borghans JA (2011). Long-term restoration of the human T-cell compartment after thymectomy during infancy: a role for thymic regeneration?. Blood.

[b18] Goronzy JJ, Weyand CM (2013). Understanding immunosenescence to improve responses to vaccines. Nat. Immunol.

[b19] Goronzy JJ, Lee WW, Weyand CM (2007). Aging and T-cell diversity. Exp. Gerontol.

[b20] Hazenberg MD, Otto SA, Cohen Stuart JW, Verschuren MC, Borleffs JC, Boucher CA, Coutinho RA, Lange JM, Rinke de Wit TF, Tsegaye A, van Dongen JJ, Hamann D, de Boer RJ, Miedema F (2000a). Increased cell division but not thymic dysfunction rapidly affects the T-cell receptor excision circle content of the naive T cell population in HIV-1 infection. Nat. Med.

[b21] Hazenberg MD, Stuart JW, Otto SA, Borleffs JC, Boucher CA, de Boer RJ, Miedema F, Hamann D (2000b). T-cell division in human immunodeficiency virus (HIV)-1 infection is mainly due to immune activation: a longitudinal analysis in patients before and during highly active antiretroviral therapy (HAART). Blood.

[b22] Hazenberg MD, Otto SA, de Pauw ES, Roelofs H, Fibbe WE, Hamann D, Miedema F (2002). T-cell receptor excision circle and T-cell dynamics after allogeneic stem cell transplantation are related to clinical events. Blood.

[b23] Hogan T, Shuvaev A, Commenges D, Yates A, Callard R, Thiebaut R, Seddon B (2013). Clonally diverse T cell homeostasis is maintained by a common program of cell-cycle control. J. Immunol.

[b24] Kogut I, Scholz JL, Cancro MP, Cambier JC (2012). B cell maintenance and function in aging. Semin. Immunol.

[b25] Kuranda K, Vargaftig J, de la Rochere P, Dosquet C, Charron D, Bardin F, Tonnelle C, Bonnet D, Goodhardt M (2011). Age-related changes in human hematopoietic stem/progenitor cells. Aging Cell.

[b26] Lazuardi L, Jenewein B, Wolf AM, Pfister G, Tzankov A, Grubeck-Loebenstein B (2005). Age-related loss of naive T cells and dysregulation of T-cell/B-cell interactions in human lymph nodes. Immunology.

[b27] Macallan DC, Wallace DL, Zhang Y, Ghattas H, Asquith B, de Lara C, Worth A, Panayiotakopoulos G, Griffin GE, Tough DF, Beverley PC (2005). B-cell kinetics in humans: rapid turnover of peripheral blood memory cells. Blood.

[b28] Macaulay R, Akbar AN, Henson SM (2013). The role of the T cell in age-related inflammation. Age (Dordr.).

[b29] McKenna RW, Washington LT, Aquino DB, Picker LJ, Kroft SH (2001). Immunophenotypic analysis of hematogones (B-lymphocyte precursors) in 662 consecutive bone marrow specimens by 4-color flow cytometry. Blood.

[b30] Michishita Y, Hirokawa M, Guo YM, Abe Y, Liu J, Ubukawa K, Fujishima N, Fujishima M, Yoshioka T, Kameoka Y, Saito H, Tagawa H, Takahashi N, Sawada K (2011). Age-associated alteration of γδ T-cell repertoire and different profiles of activation-induced death of Vδ1 and Vδ2 T cells. Int. J. Hematol.

[b31] Miller RA, Stutman O (1984). T cell repopulation from functionally restricted splenic progenitors: 10,000-fold expansion documented by using limiting dilution analyses. J. Immunol.

[b32] Montecino-Rodriguez E, Berent-Maoz B, Dorshkind K (2013). Causes, consequences, and reversal of immune system aging. J. Clin. Invest.

[b33] Naylor K, Li G, Vallejo AN, Lee WW, Koetz K, Bryl E, Witkowski J, Fulbright J, Weyand CM, Goronzy JJ (2005). The influence of age on T cell generation and TCR diversity. J. Immunol.

[b34] Ogawa T, Kitagawa M, Hirokawa K (2000). Age-related changes of human bone marrow: a histometric estimation of proliferative cells, apoptotic cells, T cells, B cells and macrophages. Mech. Ageing Dev.

[b35] Patterson BW, Zhao G, Klein S (1998). Improved accuracy and precision of gas chromatography/mass spectrometry measurements for metabolic tracers. Metabolism.

[b36] Re F, Poccia F, Donnini A, Bartozzi B, Bernardini G, Provinciali M (2005). Skewed representation of functionally distinct populations of Vgamma9Vdelta2 T lymphocytes in aging. Exp. Gerontol.

[b37] Roux A, Mourin G, Larsen M, Fastenackels S, Urrutia A, Gorochov G, Autran B, Donner C, Sidi D, Sibony-Prat J, Marchant A, Stern M, Sauce D, Appay V (2013). Differential impact of age and cytomegalovirus infection on the gammadelta T cell compartment. J. Immunol.

[b38] Sauce D, Larsen M, Fastenackels S, Roux A, Gorochov G, Katlama C, Sidi D, Sibony-Prat J, Appay V (2012). Lymphopenia-driven homeostatic regulation of naive T cells in elderly and thymectomized young adults. J. Immunol.

[b39] Saule P, Trauet J, Dutriez V, Lekeux V, Dessaint JP, Labalette M (2006). Accumulation of memory T cells from childhood to old age: central and effector memory cells in CD4(+) versus effector memory and terminally differentiated memory cells in CD8(+) compartment. Mech. Ageing Dev.

[b40] Steinmann GG, Klaus B, Muller-Hermelink HK (1985). The involution of the ageing human thymic epithelium is independent of puberty. A morphometric study. Scand. J. Immunol.

[b41] Takada K, Jameson SC (2009). Naive T cell homeostasis: from awareness of space to a sense of place. Nat. Rev. Immunol.

[b42] Vrisekoop N, den Braber I, de Boer AB, Ruiter AF, Ackermans MT, van der Crabben SN, Schrijver EH, Spierenburg G, Sauerwein HP, Hazenberg MD, de Boer RJ, Miedema F, Borghans JA, Tesselaar K (2008). Sparse production but preferential incorporation of recently produced naive T cells in the human peripheral pool. Proc. Natl Acad. Sci. USA.

[b101] Wallace DL, Zhang Y, Ghattas H, Worth A, Irvine A, Bennett AR, Griffin GE, Beverley PCL, Tough DF, Macallan DC (2004). Direct measurement of T Cell subset kinetics in vivo in elderly men and women. J Immunol.

[b43] Watson PE, Watson ID, Batt RD (1980). Total body water volumes for adult males and females estimated from simple anthropometric measurements. Am. J. Clin. Nutr.

[b44] Wertheimer AM, Bennett MS, Park B, Uhrlaub JL, Martinez C, Pulko V, Currier NL, Nikolich-Zugich D, Kaye J, Nikolich-Zugich J (2014). Aging and cytomegalovirus infection differentially and jointly affect distinct circulating T cell subsets in humans. J. Immunol.

[b45] Westera L, Drylewicz J, den Braber I, Mugwagwa T, van der Maas I, Kwast L, Volman T, van de Weg-Schrijver EH, Bartha I, Spierenburg G, Gaiser K, Ackermans MT, Asquith B, de Boer RJ, Tesselaar K, Borghans JA (2013a). Closing the gap between T-cell life span estimates from stable isotope-labeling studies in mice and humans. Blood.

[b46] Westera L, Zhang Y, Tesselaar K, Borghans JA, Macallan DC (2013b). Quantitating lymphocyte homeostasis in vivo in humans using stable isotope tracers. Methods Mol. Biol.

[b47] Westermann J, Pabst R (1990). Lymphocyte subsets in the blood: a diagnostic window on the lymphoid system?. Immunol. Today.

