# Supplementary material for: Lymphocyte maintenance during healthy aging requires no substantial alterations in cellular turnover
Source: Aging Cell. 2015 Jan 28;14(2):219–27. doi: 10.1111/acel.12311 (PMC4364834; doi:10.1111/acel.12311)
Supplement: Supplementary file 1 [file acel0014-0219-sd1.docx]

**Supporting Information**

**Lymphocyte maintenance during healthy aging requires no substantial alterations in cellular turnover**

Liset Westera^1#^, Vera van Hoeven^1#^, Julia Drylewicz^1,2^, Gerrit Spierenburg^1^, Jeroen F van Velzen^1^, Rob J de Boer^2^, Kiki Tesselaar^1¶^, José AM Borghans^1¶^*

^1^Laboratory for Translational Immunology, Department of Immunology, University Medical Center Utrecht, Lundlaan 6, 3584 EA, Utrecht, The Netherlands

^2^Theoretical Biology & Bioinformatics, Department of Biology, Utrecht University, Padualaan 8, 3584 CH, Utrecht, The Netherlands

^#,¶^ These authors contributed equally to this work

*corresponding author: J.Borghans@umcutrecht.nl

**Supporting Methods**

***Mathematical modeling.*** To control for changing levels of ^2^H in body water over the course of the experiment, ^2^H enrichment in urine was fitted with a simple label enrichment/decay curve for each individual:

during label intake (t ≤ τ): $U(t) = f(1-\text{e}^{-\delta t})+\beta\text{e}^{-\delta t}$ (Equation 1a)

after label intake (t > τ): $U(t) = \left[f(1-\text{e}^{-\delta\tau})+\beta\text{e}^{-\delta\tau}\right] \text{e}^{-\delta(t-\tau)}$ (Equation 1b)

as described previously (Vrisekoop *et al*., 2008), where U(t) represents the fraction of ^2^H_2_O in plasma at time t (in days), f is the fraction of ^2^H_2_O in the drinking water, labeling was stopped at t = τ days, δ represents the turnover rate of body water per day, and β is the plasma enrichment attained after the boost of label by the end of day 0. We incorporated these best fits when analyzing the enrichment in the different cell populations. Up- and delabeling of the granulocyte population of each individual was analyzed as described previously (Vrisekoop *et al*., 2008), to estimate the maximum level of label intake that cells could possibly attain. The label enrichment data of all cell subsets were subsequently scaled by the granulocyte asymptote of each individual.

Labeling data of the different leukocyte subsets were fitted with a mathematical model that allowed for kinetic heterogeneity between cells of the same population. Each kinetic subpopulation i was modelled to contain a fraction α_i_ of cells with turnover rate p_i_. Because the population sizes hardly changed, we considered a steady state for each kinetic subpopulation (i.e., production equals loss), and label enrichment of adenosine in the DNA of each subpopulation i was modelled by the following differential equation:

$\frac{\text{d}l_{i}}{\text{d}t} = p_{i}cU(t)\alpha_{i}A-p_{i}l_{i}$ (Equation 2a)

where l_i_ is the total amount of labeled adenosine in the DNA of subpopulation i and A is the total amount of adenosine in the cell population under investigation, c is an amplification factor that needs to be introduced because the adenosine deoxyribose (dR) moiety contains multiple hydrogen atoms that can be replaced by deuterium (Vrisekoop *et al*., 2008), and p_i_ is the average turnover rate of subpopulation i. Basically, labeled adenosines in subpopulation i are gained when a deuterium atom is incorporated with probability cU(t) in the DNA of cells that replicate at rate p_i_, and they are lost when cells of subpopulation i are lost at rate p_i_. For naive T cells this replication may occur both in the periphery and in the thymus. Scaling this equation by the total amount of adenosine in the DNA of subpopulation i, i.e., defining L_i_ = l_i_/(α_i_A), yields

$\frac{\text{d}L_{i}}{\text{d}t} = p_{i}cU(t)-p_{i}L_{i}$ (Equation 2b)

throughout the up- and delabeling period, where L_i_ represents the fraction of labeled adenosine dR moieties in the DNA of subpopulation i. The corresponding analytical solutions are

$L_{i}(t) = \frac{c}{\delta-p_{i}}\left[\delta f(1-\text{e}^{-p_{i} t})-p_{i}f(1-\text{e}^{-\delta t})+\beta p_{i}(\text{e}^{-p_{i} t}-\text{e}^{-\delta t})\right]$ (Equation 3a)

during label intake (t ≤ τ ), and

$L_{i}(t) = \frac{c}{\delta-p_{i}}\left[\delta f(\text{e}^{-p_{i} (t-\tau)}-\text{e}^{-p_{i} t})-p_{i}f(\text{e}^{-\delta(t-\tau)}-\text{e}^{-\delta t})+\beta p_{i}(\text{e}^{-p_{i} t}-\text{e}^{-\delta t})\right]$ (Equation 3b)

after label intake (t > τ).

The fraction of labeled DNA in the total T-cell population under investigation was subsequently derived from $L (t)= \sum\alpha_{i}L_{i}(t)$, and the average turnover rate p was calculated from $p = \sum\alpha_{i}p_{i}$.

Because all enrichment data were expressed as fractions, labeling data were arcsin(sqrt) transformed before the mathematical model was fitted to the data. We followed a stepwise selection procedure to determine the number of kinetically different subpopulations to include in the model, adding a new kinetically different subpopulation into the model until the average turnover rate was no longer significantly changed (Westera et al., 2013). For populations that appeared to behave kinetically homogeneously, the fitting procedure set the contribution of the extra subpopulation(s) to zero. Average turnover rates p of different leukocyte populations were estimated by fitting the enrichment data for each individual and average lifespans were calculated as 1/p. The enrichment data were also fitted using mixed-effects models to illustrate the difference in dynamics at the group level (Fig. 3A). The average turnover rates estimated using mixed-effects models are not reported in the manuscript and did not differ from the ones we estimated by doing individual fitting. The labeling curves of memory CD4^+^ and CD8^+^ T-cells, as well of memory and natural effector B-cells were significantly better described by a model including two kinetically different subpopulations while the other leukocyte populations required only one.

Daily thymic output was calculated as described previously (den Braber *et al*., 2012), **based on a model describing naive T-cells and TREC dynamics in the periphery at a**cellular level**described by**Hazenberg *et al*., (2000). Briefly, the total production of naive CD4^+^ T cells per day was first calculated as (the average turnover rate *p*) x (the absolute number of naive CD4^+^ T cells per liter blood) x (5 liter blood) x 50, assuming that 2% of lymphocytes reside in the blood (Westermann & Pabst, 1990) . The total daily naive CD4^+^ T-cell production was multiplied by the normalized naive CD4^+^ T-cell TREC content (normalized using TREC contents of single positive CD4^+^ thymocytes) to estimate the daily thymic output in cells/day. The peripheral T-cell division rate per day was obtained by subtracting daily thymic output from total daily production and dividing this value by the absolute number of naive CD4^+^ T cells**. The model used to calculate T-cell turnover (from deuterium labeling data) is based on the same “basic” model describing naive T-cell dynamics in the periphery but it has a different structure than the TREC model. Firstly, it describes incorporation and loss of deuterium at the DNA level. Secondly, it does not contain an explicit term for the thymic output because at the DNA level, one cannot distinguish between deuterium incorporated during cell division in the thymus or during peripheral T-cell division. Therefore, the model used to calculate T-cell turnover describes the dynamics of the sum of thymic output and peripheral T-cell division. Because both models are derived from the same "basic model" one can use the combined results to calculate peripheral T-cell division.**

**Supporting Figures**

**
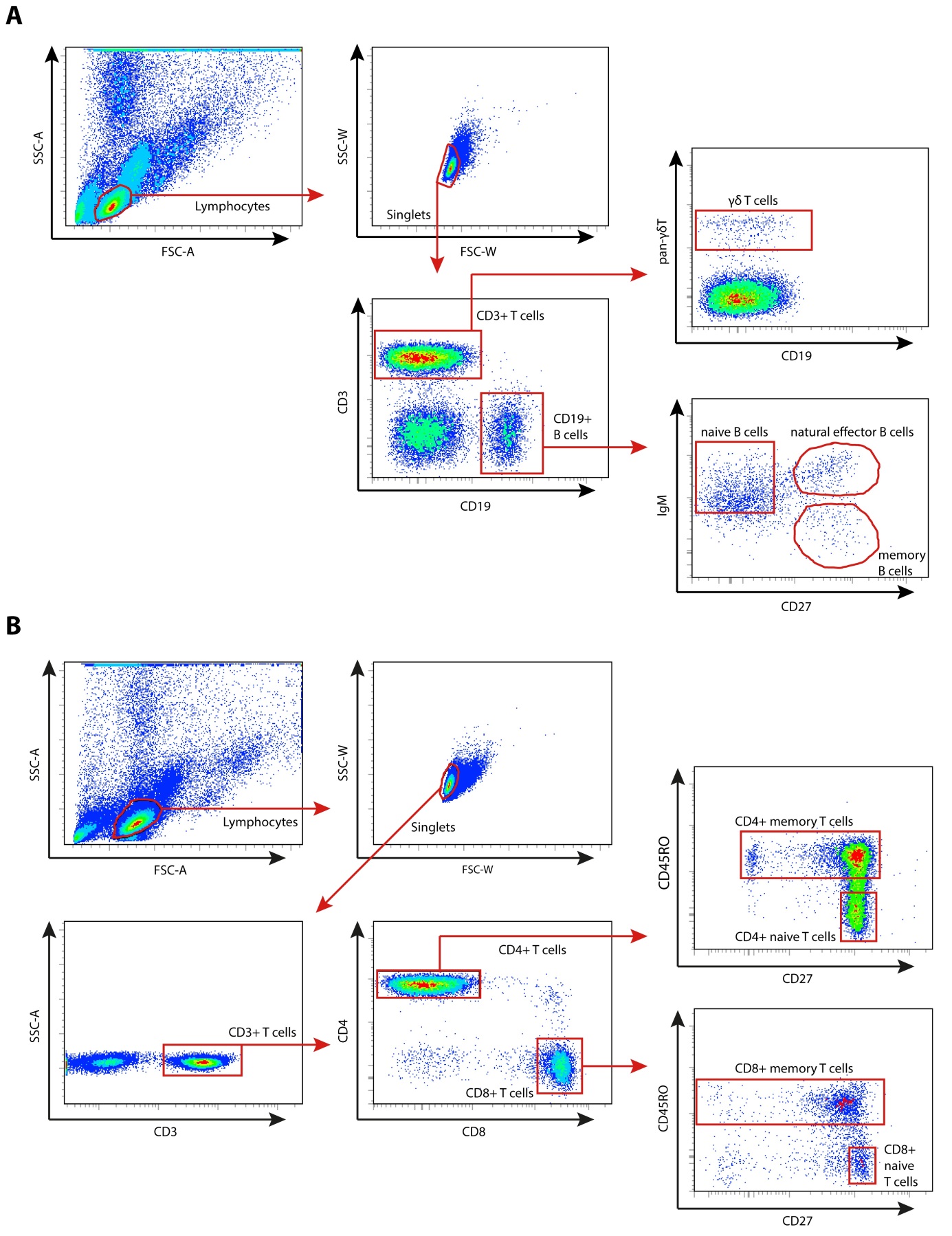
**

**Fig S1.** Sort gating strategy. Strategy for sorting B cells and γδ T cells (A) and for naive and memory CD4^+^ and CD8^+^ T cells (B).

**
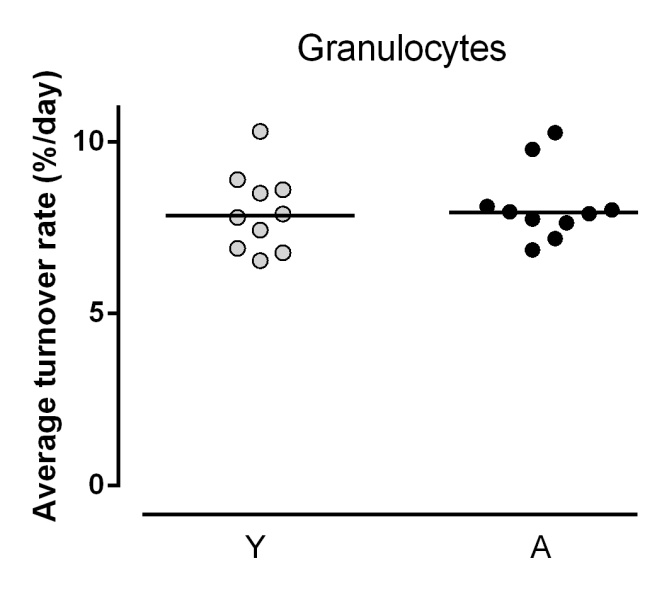
**

**Fig. S2** Estimated average turnover rates of granulocytes in young and elderly individuals. Estimates of the average turnover rate of granulocytes in young (gray symbols) and aged (black symbols) individuals. Horizontal lines represent median values.

**
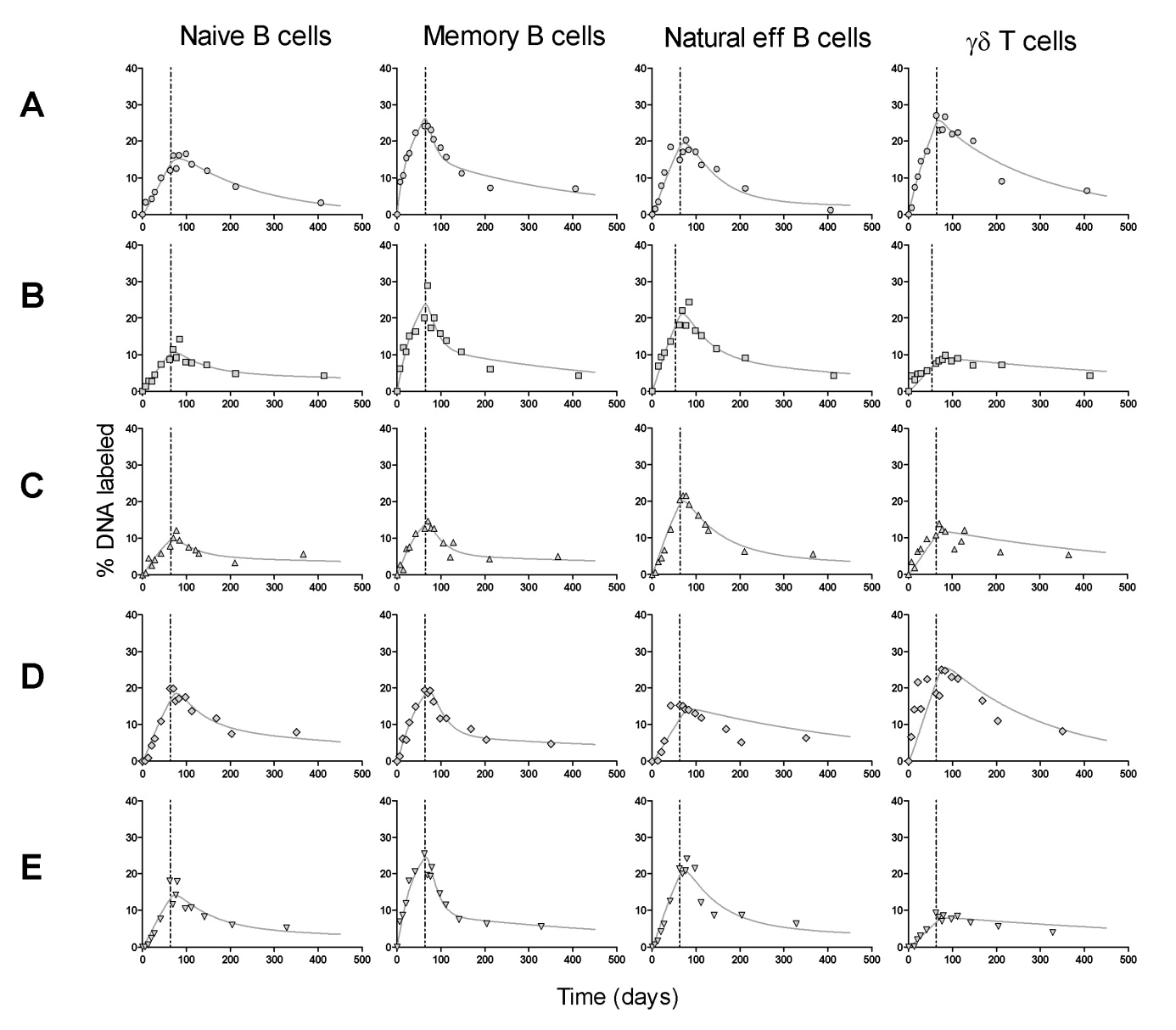
 Fig. S3**. Best fits of ^2^H enrichment in B-cell subsets and in γδ T cells in young individuals. Best ﬁts of the multi-exponential model to the enrichment in naive, memory, and natural effector (Natural eff) B cells, and γδ T cells in the five young individuals (A-E). Label enrichment in the DNA was scaled between 0 and 100% by normalizing for the maximum enrichment in granulocytes (see Supporting Methods). The end of ^2^H_2_O administration is marked by a dashed vertical line.

**
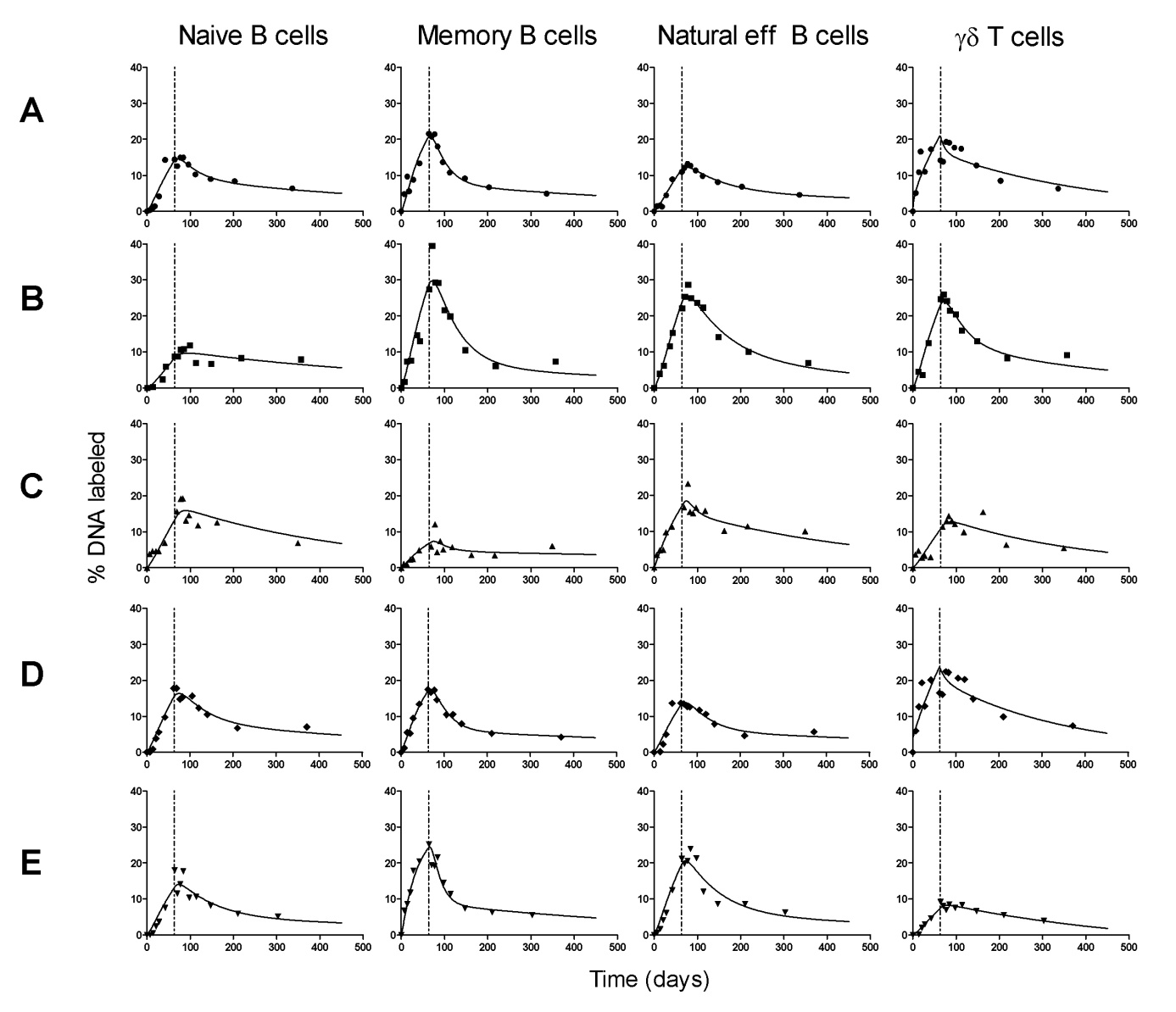
**

**Fig. S4** Best fits of ^2^H enrichment in B-cell subsets and in γδ T cells in elderly individuals. Best ﬁts of the multi-exponential model to the enrichment in naive, memory, and natural effector (Natural eff) B cells, and γδ T cells in the five aged individuals (A-E). Label enrichment in the DNA was scaled between 0 and 100% by normalizing for the maximum enrichment in granulocytes (see Supporting Methods). The end of ^2^H_2_O administration is marked by a dashed vertical line.

**
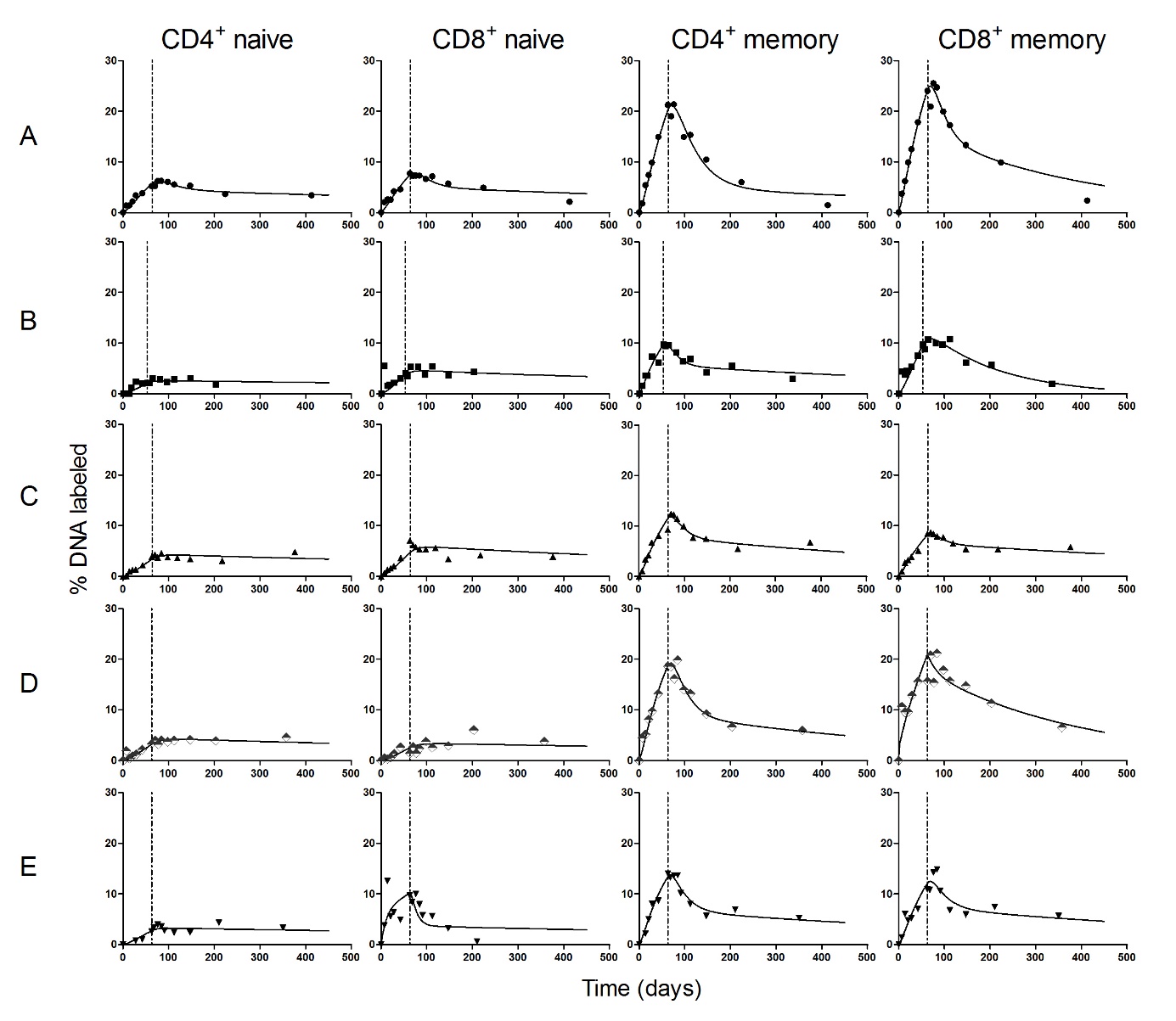
**

**Fig. S5** Best fits of ^2^H enrichment in T-cell subsets in elderly individuals. Best ﬁts of the multi-exponential model to the enrichment in naive and memory CD4^+^ and CD8^+^ T cells in the five aged individuals. Label enrichment in the DNA was scaled between 0 and 100% by normalizing for the maximum enrichment in granulocytes (see Supporting Methods). The end of ^2^H_2_O administration is marked by a dashed vertical line. Enrichment data of the corresponding subsets in young individuals were previously published (Vrisekoop *et al*., 2008; Westera *et al*., 2013). (D) This elderly male tested seropositive for CMV.


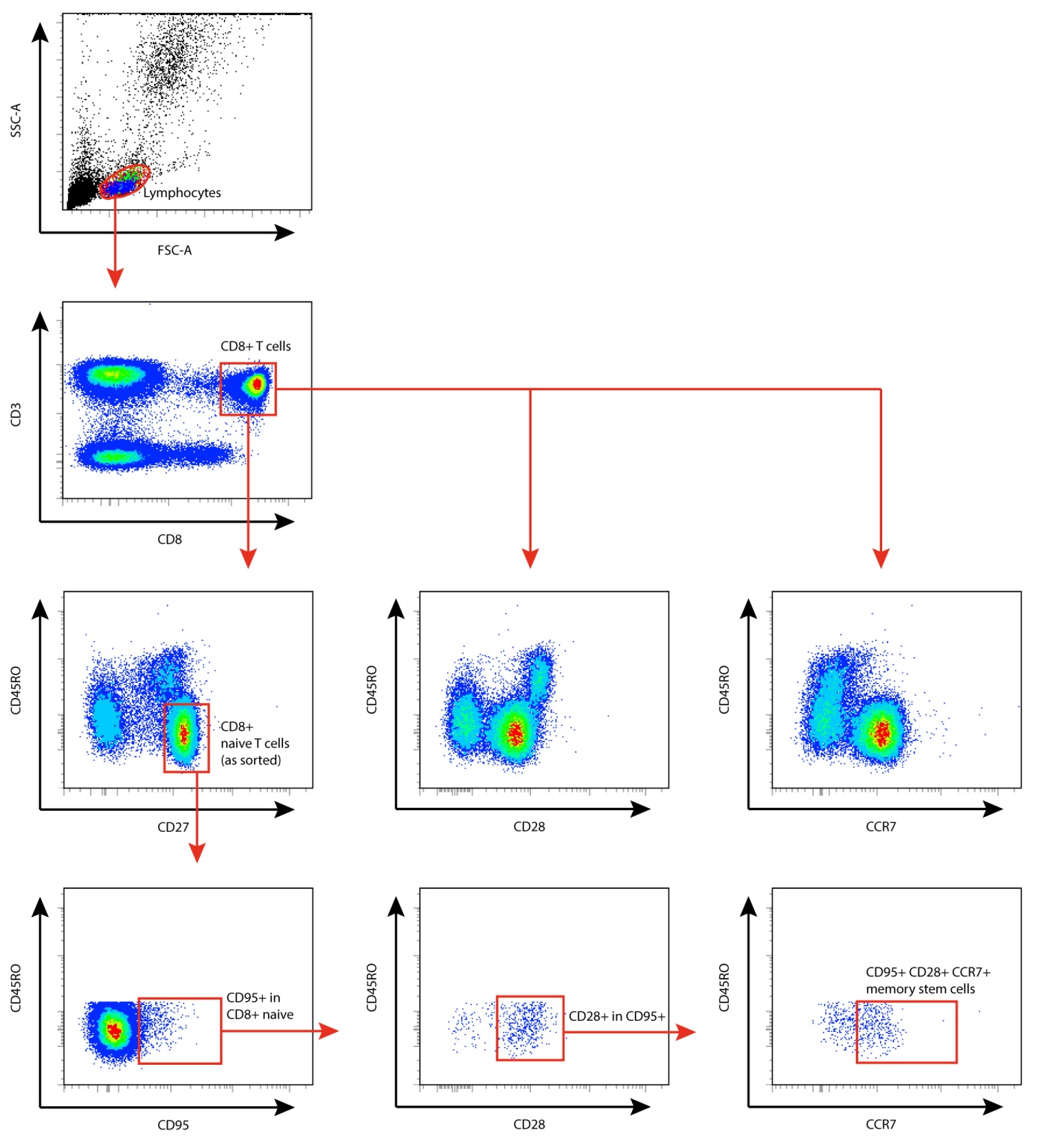


Fig S6. Composition of the CD95^+^ fraction of the naive CD8^+^ T-cell pool. Representative density dot plots of flow cytometric analysis of the CD95^+^ fraction present in sorted naive CD8^+^ T cells. This specific analysis was performed for 3 young and 8 aged participants of the heavy water labeling study.

**
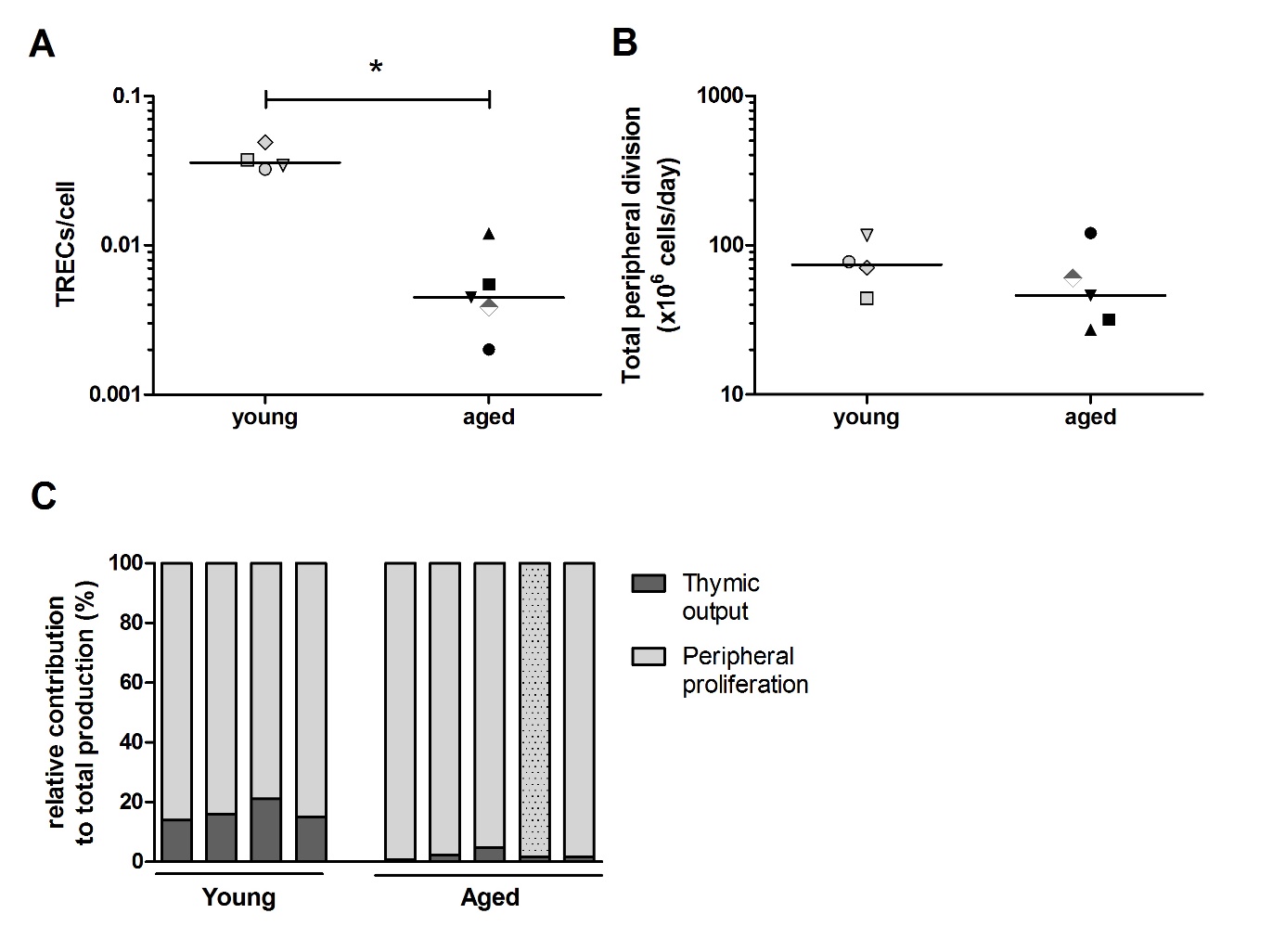
**Fig. S7 TREC content and total peripheral division of naive CD4^+^ T cells in young and elderly individuals. (A) The number of TRECs/cell was determined in naive CD4^+^ cells of the five elderly individuals enrolled in this study for analysis of T cell subsets, and compared with the TREC content that was determined for four young individuals in the previous T-cell ^2^H_2_O study (den Braber *et al*., 2012). Due to limited material, TREC contents could not be reliably measured for naive CD8^+^ T cells. The asterisk marks a significant difference (p-value<0.05). (B) Estimated total peripheral division in cells per day, obtained by subtracting the estimated daily thymic output from the total naive CD4^+^ T-cell production. (A+B) The elderly male who tested seropositive for CMV is depicted by a semi-filled diamond. (C) Relative contributions of thymic output (dark gray bars) and peripheral T-cell division (light gray bars) to the total production of naive CD4^+^ T cells. The elderly male who tested seropositive for CMV is indicated by a dotted bar.


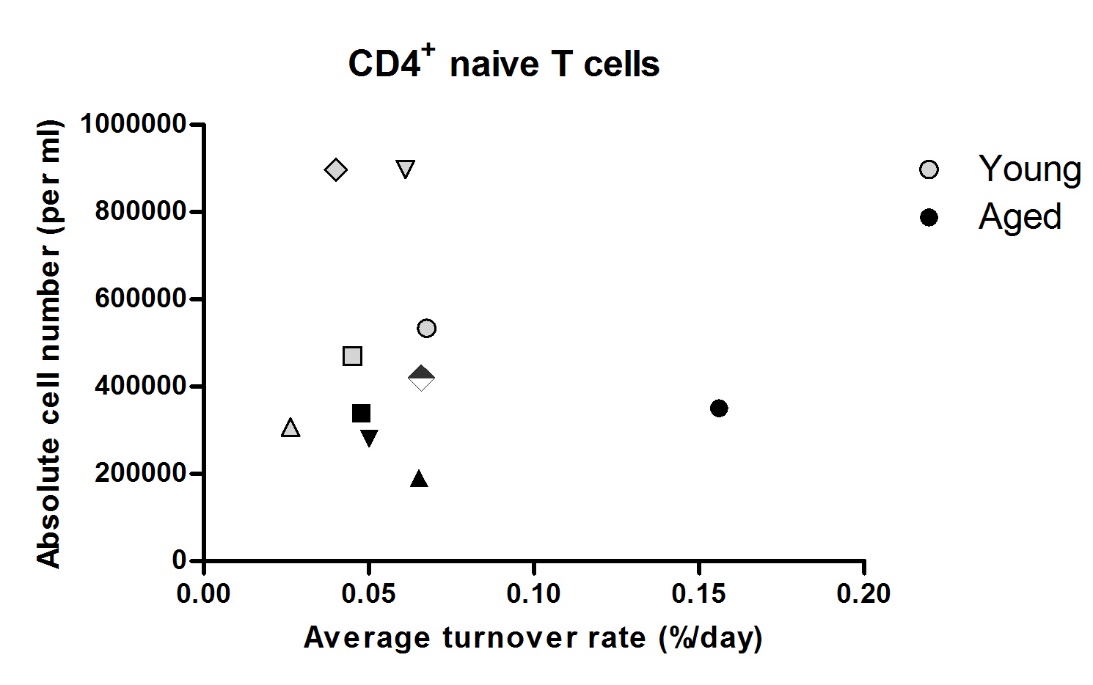


Fig S8. No correlation between the absolute number and the turnover rate of CD4^+^ naive T cells. Absolute CD4^+^ naive T-cell counts from young and aged individuals (Fig. 1C) plotted against the corresponding CD4^+^ naive T-cell turnover rate (Fig. 2C). Within the naive CD4^+^ T-cell population, there is no significant correlation between the absolute cell count and average turnover rate (p-value=0.95). The elderly male who tested seropositive for CMV is indicated by a semi-filled diamond.

**References**

1. den Braber I, Mugwagwa T, Vrisekoop N, Westera L, Mogling R, de Boer AB, Willems N, Schrijver EH, Spierenburg G, Gaiser K, Mul E, Otto SA, Ruiter AF, Ackermans MT, Miedema F, Borghans JA, de Boer RJ, Tesselaar K (2012) Maintenance of peripheral naive T cells is sustained by thymus output in mice but not humans. *Immunity.* **36**, 288-297.

2. Hazenberg MD, Otto SA, Cohen Stuart JW, Verschuren MC, Borleffs JC, Boucher CA, Coutinho RA, Lange JM, Rinke de Wit TF, Tsegaye A, van Dongen JJ, Hamann D, de Boer RJ, Miedema F (2000) Increased cell division but not thymic dysfunction rapidly affects the T-cell receptor excision circle content of the naive T cell population in HIV-1 infection. *Nat.Med.* **6**, 1036-1042.

3. Vrisekoop N, den Braber I, de Boer AB, Ruiter AF, Ackermans MT, van der Crabben SN, Schrijver EH, Spierenburg G, Sauerwein HP, Hazenberg MD, de Boer RJ, Miedema F, Borghans JA, Tesselaar K (2008) Sparse production but preferential incorporation of recently produced naive T cells in the human peripheral pool. *Proc.Natl.Acad.Sci.U.S.A* **105**, 6115-6120.

4. Westera L, Drylewicz J, den Braber I, Mugwagwa T, van der Maas I, Kwast L, Volman T, van de Weg-Schrijver EH, Bartha I, Spierenburg G, Gaiser K, Ackermans MT, Asquith B, de Boer RJ, Tesselaar K, Borghans JA (2013) Closing the gap between T-cell life span estimates from stable isotope-labeling studies in mice and humans. *Blood* **122**, 2205-2212.

5. Westermann J, Pabst R (1990) Lymphocyte subsets in the blood: a diagnostic window on the lymphoid system? *Immunol.Today* **11**, 406-410.
